# Rilpivirine analogs potently inhibit drug-resistant HIV-1 mutants

**DOI:** 10.1186/s12977-016-0244-2

**Published:** 2016-02-16

**Authors:** Steven J. Smith, Gary T. Pauly, Aamir Akram, Kevin Melody, Ganesha Rai, David J. Maloney, Zandrea Ambrose, Craig J. Thomas, Joel T. Schneider, Stephen H. Hughes

**Affiliations:** HIV Drug Resistance Program, National Cancer Institute-Frederick, National Institutes of Health, Frederick, MD USA; Chemical Biology Laboratory, National Cancer Institute-Frederick, National Institutes of Health, Frederick, MD USA; Department of Infectious Diseases and Microbiology, Graduate School of Public Health, University of Pittsburgh, Pittsburgh, PA USA; Division of Infectious Diseases, Department of Medicine, School of Medicine, University of Pittsburgh, Pittsburgh, PA USA; NIH Chemical Genomics Center, National Center for Advancing Translational Sciences, NIH, 9800 Medical Center Drive, Bethesda, MD 3370 USA

**Keywords:** HIV-1, Nonnucleoside reverse transcriptase inhibitors, Rilpivirine, Antiviral activity, Resistance, Analogs, Susceptibility, Binding pocket, Doravirine

## Abstract

**Background:**

Nonnucleoside reverse transcriptase inhibitors (NNRTIs) are a class of antiretroviral compounds that bind in an allosteric binding pocket in HIV-1 RT, located about 10 Å from the polymerase active site. Binding of an NNRTI causes structural changes that perturb the alignment of the primer terminus and polymerase active site, preventing viral DNA synthesis. Rilpivirine (**RPV**) is the most recent NNRTI approved by the FDA, but like all other HIV-1 drugs, suboptimal treatment can lead to the development of resistance. To generate better compounds that could be added to the current HIV-1 drug armamentarium, we have developed several **RPV** analogs to combat viral variants that are resistant to the available NNRTIs.

**Results:**

Using a single-round infection assay, we identified several **RPV** analogs that potently inhibited a broad panel of NNRTI resistant mutants. Additionally, we determined that several resistant mutants selected by either **RPV** or Doravirine (**DOR**) caused only a small increase in susceptibility to the most promising **RPV** analogs.

**Conclusions:**

The antiviral data suggested that there are **RPV** analogs that could be candidates for further development as NNRTIs, and one of the most promising compounds was modeled in the NNRTI binding pocket. This model can be used to explain why this compound is broadly effective against the panel of NNRTI resistance mutants.

**Electronic supplementary material:**

The online version of this article (doi:10.1186/s12977-016-0244-2) contains supplementary material, which is available to authorized users.

## Background

HIV-1 reverse transcriptase (RT) is a target for many drugs used in highly active antiretroviral therapy (HAART) to treat HIV-1 infections [1]. RT has two enzymatic activities: (1) a DNA polymerase that can copy either an RNA or a DNA template and (2) an RNase H that degrades RNA if and only if the RNA is part of a RNA: DNA hybrid. Although there are, as yet, no drugs that target the RNase H of HIV-1 RT, there are two classes of drugs that target the DNA polymerase. The first class consists of inhibitors that are analogs of normal nucleosides used to synthesize DNA (nucleoside reverse transcriptase inhibitors, NRTIs). All of the FDA-approved NRTIs lack the 3′ –OH present on the deoxyribose of normal nucleosides. When an NRTI is incorporated into the growing viral DNA strand, it acts as a chain terminator. The second class consists of inhibitors that bind in a small hydrophobic pocket ~10 Å from the polymerase active site (non-nucleoside reverse transcriptase inhibitors, NNRTIs) [2–4]. The NNRTI binding pocket is closed in the absence of a bound NNRTI [5]. The NNRTI binding pocket is formed by the following residues: L100, K101, K103, V106, V179, Y181, Y188, G190, F227, W229, L234, P236, and Y318. The binding pocket lies underneath the bound double-stranded nucleic acid substrate. Binding of an NNRTI distorts RT, which affects the alignment of the primer terminus and the polymerase active site, blocking the chemical step of viral DNA synthesis [6–8]. There currently are sixteen drugs that target RT that have been approved by the FDA for the treatment of HIV-1 infections; eleven are NRTIs and five are NNRTIs. The five approved NNRTI drugs are: nevirapine (**NVP**, Viramune), delavirdine (**DLV**, Rescriptor), efavirenz (**EFV**, Sustiva), etravirine (**ETR**, Intelence), and rilpivirine (**RPV**, Endurant,). While NNRTIs effectively block the replication of WT HIV-1, NNRTI-resistant mutants can emerge during treatment, many of which cause cross-resistance among the approved NNRTIs.

There are two primary factors that contribute to the emergence of resistance to NNRTIs: (1) HIV-1 RT can tolerate a wide range of sequences in and around the NNRTI binding pocket and (2) there is extensive HIV-1 genetic variation [9, 10]. Although most HIV-infected individuals are initially infected with a single virion, HIV-1 variants arise rapidly due to high viral loads in HIV-1 infected patients, which leads to the infection of large numbers of cells, the rapid turnover of these infected cells, and to errors made during HIV-1 replication [10–12]. Ultimately, error prone replication creates the mutations that enable HIV-1 to develop resistance against antiretroviral drugs.

A number of drug resistant mutants were selected in HIV-infected individuals by the first generation NNRTIs (**NVP**, **EFV**, and **DLV**). Resistance mutations are commonly seen in the residues that surround the NNRTI binding pocket, including: L100I, K103N, V106A, Y181C, Y188L, and H221Y. These mutations alter the geometry of the NNRTI binding pocket in ways that interfere with the binding of NNRTIs. The first generation inhibitors were rigid and bulky compounds that were particularly vulnerable to the effects of resistance mutations. The second generation NNRTIs, **ETR** and **RPV**, were designed to be less bulky and more flexible, and are better able to adapt to the changes in the NNRTI binding pocket caused by resistance mutations. This allows these newer NNRTIs to effectively inhibit both WT HIV-1 and a number of drug resistant variants [13, 14]. However, when these drugs were tested in clinical trials, patients who were on an **ETR** containing regimen were found to have a combination of mutations including: V90I, A98G, L100I, K101E/P, V106I, V179D/F, Y181C/I/V, and G190A/S [15]. A variety of mutations including K101E/P, E138A/G/K/Q/R, Y181C/I/V, and M230L have been shown to be associated with a decrease in susceptibility to **RPV** and have been detected in clinical samples from HIV-1-infected individuals treated with **RPV** [16]. Individuals who failed in a clinical trial involving therapy with **RPV** and NRTIs had mutations at positions E138K and M184I/V; the M184V mutation was likely selected by **FTC**, an NRTI often included in HAART regimens [17, 18].

Because development of resistance can occur with all of the available NNRTIs, there are ongoing efforts to develop NNRTIs that extend and improve the effectiveness of the previously identified compounds. For example, doravirine (**DOR**) is a new NNRTI in late stage clinical trials [19]. Although it selects for resistance, the mutations that reduce the potency of **DOR** do not, for the most part, overlap with mutations that reduce the potency of **RPV** [20]. As an alternative approach, we are testing whether **RPV** analogs have better resistance profiles than the parent compound.

Several of our **RPV** analogs are broadly effective against HIV-1 mutants. One compound (**11**) was particularly effective in inhibiting the replication of mutants that have reduced susceptibility to **RPV** and **DOR**, suggesting that it has the potential to become a clinically useful NNRTI.

## Results and discussion

### Susceptibility of mutant HIV-1 to RPV analogs

We previously determined the ability of several of our **RPV** analogs to inhibit infection of WT HIV-1 or mutants that contain several well-known NNRTI-resistant mutations (L100I, K103N, E138K, Y181C, Y188L, H221Y, and K103N/Y181C) using a single-round infection luciferase assay [21]. As shown in Figs. 3 and 4 of that aforementioned study, **RPV** and several analogs (**6**, **7**, **8**, **9**, **11**, **12**, **13**, **14**, **15**, **16**, **17**, **21**, and **27**) had potent antiviral activities against both WT HIV-1 and several of the resistant mutants L100I, K103N, Y181C, and H221Y (all IC_50_s < 7 nM). The E138K mutant caused only a small drop in the IC_50_s for most of the compounds (<5 nM); however there was a greater loss in susceptibility to **12** (15 nM ± 1.2). The resistant mutant Y188L showed modest drop in susceptibility to **8**, **9**, **11**, **12**, **14**, **15**, **16**, and **17** (IC_50_s ranged from 12 to 64 nM), whereas compounds **6**, **7**, **13**, **21**, and **27** showed a modest loss of potency against this resistant mutant (all <10 nM). The NNRTI resistant double mutant K103N/Y181C caused a substantial decrease in susceptibility to RPV analogs **6**, **8**, **9**, **13**, **21**, and **27** (ranged from 25 to 100 nM), whereas this mutant caused a minor loss in susceptibility to **RPV**, **7**, **11**, and **13** (<5 nM).

**Fig. 1:**
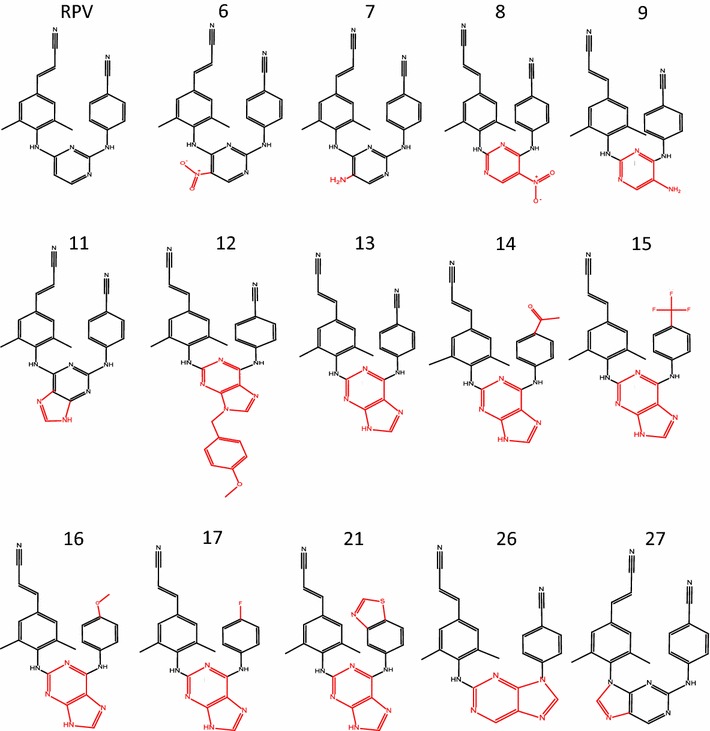
Chemical Structures of the RPV Analogs. Chemical structures of **RPV** and the **RPV** analogs. The parts of the analogs that differ from **RPV** are indicated in *red*

Because several of our **RPV** analogs were active against the first set of NNRTI resistant mutants, we tested the antiviral activities of **RPV** and the most promising analogs **6**, **7**, **8**, **9**, **11**, **12**, **13**, **14**, **15**, **16**, **17**, **21**, **26**, and **27** against a much broader panel of resistant mutants. These compounds are structural homologs of **RPV** (Fig. 1) that have the following differences: the addition of an exocyclic moiety at position 5 (**6**, **7**) or the central pyrimidine moiety is in a “flipped” conformation relative to **RPV** (**8**,**9**), a substitution of the central pyrimidine ring with a 2,6-purine ring system (**11**) or this same 2,6-purine ring system in the “flipped” conformation with or without a protecting group (**12**, **13**) or this same 2,6-purine ring system with additional modifications on the benzyl moiety (**14**, **15**, **16**, **17**, **21**), or a substitution of central pyrimidine ring with a 2,9-purine ring system (**27**) or this ring system in a “flipped” orientation (**26**).

**Fig. 2:**
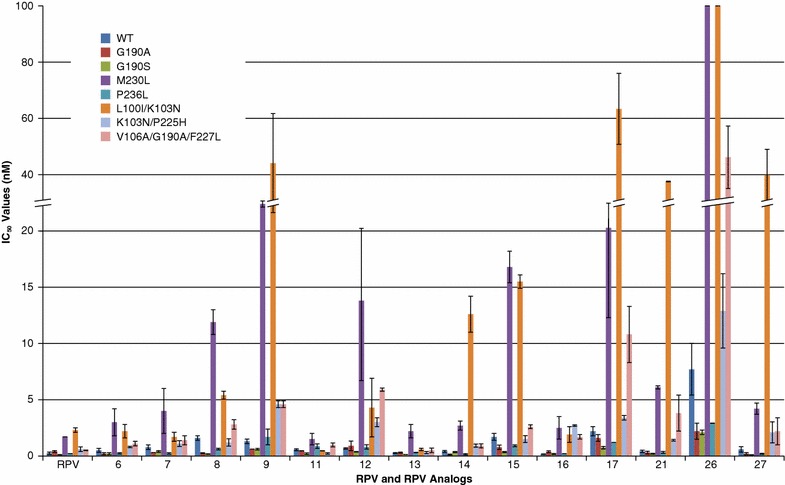
Antiviral activities of **RPV** and the **RPV** analogs against well-characterized NNRTI resistant mutants. The IC_50_ values of **RPV** and the **RPV** analogs were determined using a vector that carries WT RT and several well-known NNRTI resistant mutants in a single round infection assay. *Error bars* represent thestandard deviations of independent experiments, n = 4. The IC_50_ values of the graph are at a maximum value of 100 nM and the IC_50_ value of **26** against the M230L and L100I/K103N resistant mutants was beyond the point of detection in our single round infection assay, >100 nM. To better illustrate the lower IC_50_ values, the *Y*-axis is broken from 20 to 40 nM and also shown in the *corresponding bars*

To determine the ability of these **RPV** analogs to inhibit a wider range of NNRTI mutants, we tested their antiviral activity in a single-round infectivity assay against: G190A, G190S, M230L, P236L, L100I/K103N, K103N/P225H, and V106A/G190A/F227L (Fig. 2; Additional file 1: Table S1). Compound **26**, which has a central 2,9-purine ring system in the “flipped” orientation, had poor activities in the initial study and was included with the more promising compounds to see if there was obvious complementarity in terms of which mutants the compounds would inhibit. **RPV** and the analogs **6**, **7**, **8**, **9**, **11**, **12**, **13**, **14**, **15**, **16**, **17**, **21**, **26**, and **27** all potently inhibited the replication of WT HIV-1. The IC_50_ values in the single round assay were, for the most part, low nanomolar against the WT (all <2.2 nM; except for **26**, which was 7.7 nM ± 2.3), and against several of the RT mutants: G190A (all <1.6 nM; except for **26**, which was 2.2 nM ± 0.71), G190S (all <1 nM; except for **26**, which was 2.1 nM ± 0.2), P236L RT (all <1.7 nM; except for **26**, which was 2.91 nM ± 0.007), and K103N/P225H (all <4.6 nM; except for **26**, which was 12.9 nM ± 3.3). In the crystal structure of HIV-1 RT bound to **RPV**, position 190 is located at the back of the NNRTI binding pocket and is >6 Å away from **RPV**. When we modeled the binding of the **RPV** analogs in the NNRTI binding pocket, the distances of the compounds to position 190 were similar to **RPV** (Figs. 6, 7), which explains why mutations at 190 do not interfere with the binding of this family of inhibitors. The models show that the **RPV** analogs are 3–4 Å from amino acids 225 and 236, which reside in the depths of the NNRTI binding pocket (Figs. 6, 7). HIV-1 containing the M230L mutation was susceptible to **RPV** and to the analogs **6**, **7**, **11**, **13**, **14**, **16**, **21**, and **27** (all IC_50_s < 6.1 nM), but showed a modest decrease in susceptibility to analogs **8** (11.9 nM ± 1.1), **9** (29.6 nM ± 1.1), **12** (13.8 nM ± 7.1), **15** (16.8 nM ± 1.4), and **17** (21.1 nM ± 8.8), while compound **26** was not effective in our assay (IC_50_ > 100 nM, above the limit of detection in our assay). The antiviral results suggest that most of the **RPV** analogs with a central purine scaffold and a benzonitrile moiety identical to the one on **RPV**, or a similar moiety that lacks the fluoride modification, can successfully inhibit viruses with the M230L mutation in RT. **RPV** analogs with modifications at position 5 on the pyrimidine scaffold, with the scaffold in the same orientation as **RPV**, are also capable of inhibiting the M230L variant. The NNRTI resistant double mutant L100I/K103N posed a problem for some of the **RPV** analogs. Both compounds **14** (12.6 nM ± 1.6) and **15** (15.5 nM ± .6) showed minor reductions in sensitivity to L100I/K103N, while compounds **9** (44.1 nM ± 17.6), **17** (63.4 nM ± 12.6), **21** (37.6 nM ± 0.14), and **27** (39.8 nM ± 9.2) had a significant drop in sensitivity and **26** was beyond the limits of detection using our assay (>100 nM). However, **RPV** and analogs **6**, **7**, **8**, **11**, **12**, **13**, and **16**, showed strong antiviral activities (all <5.4 nM) against L100I/K103N. These data illustrate the importance of preserving the benzonitrile group as one of the arylamines. The problems associated with the K103N mutation might restrict the range of functional groups on the inhibitors that can be used at this position [22].

### Susceptibility of mutants selected by DOR to inhibition by RPV analogs

**Fig. 3:**
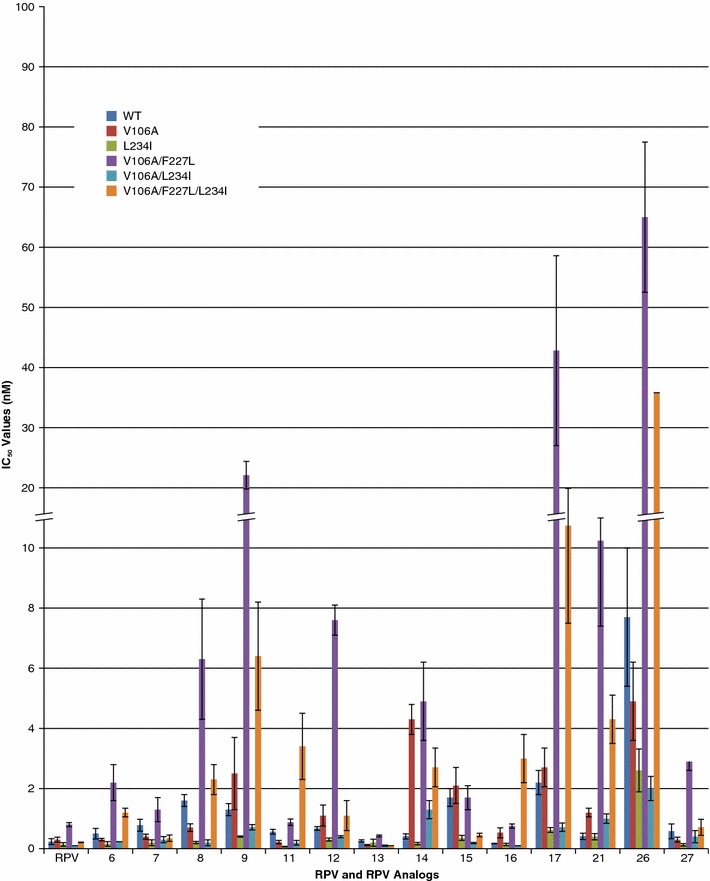
Antiviral Activities of **RPV** and **RPV** analogs against mutants selected by **DOR** in cell culture. **RPV** and the **RPV** analogs were tested against several mutants selected by DOR in cell culture. The IC_50_ values for **RPV** and the **RPV** analogs were measured using a single round infection assay. *Error bars* represent the standard deviations of independent experiments, n = 4. The IC_50_ values of the graph are at a maximum value of 100 nM. To better illustrate the lower IC_50_ values, the *Y*-axis is broken from 10 to 20 nM and also shown in the *corresponding bars*

**DOR** is a new NNRTI currently undergoing evaluation in Phase III clinical trials [19]. **DOR** was able to inhibit the replication of several well-known NNRTI-resistant mutants but readily selected resistant mutants in in vitro experiments [20]. V106A was the major variant selected in vitro and it was often accompanied by additional mutations, such as F227L, L234I, and F227L/L234I. We screened a larger panel of NNRTI-resistant mutants for their susceptibility to **DOR** and **RPV** (unpublished observations). **DOR** and **RPV** appear to differ in their susceptibility to NNRTI resistance mutations. We screened the **RPV** analogs against the mutants that were selected by DOR: V106A, 234I, V106A/F227L, V106/L234I, and V106A/F227L/L234I (Fig. 3; Additional file 1: Table S2). All 5 of these mutants were effectively inhibited by **RPV** and **6**, **7**, **8**, **9**, **11**, **12**, **13**, **14**, **15**, **16**, **17**, **21**, and **27** (all of the tested compounds had IC_50_s < 5 nM except **26**; 7.7 ± 2.3).

### Susceptibility of viruses carrying RPV-resistance mutations to the RPV analogs

**Fig. 4:**
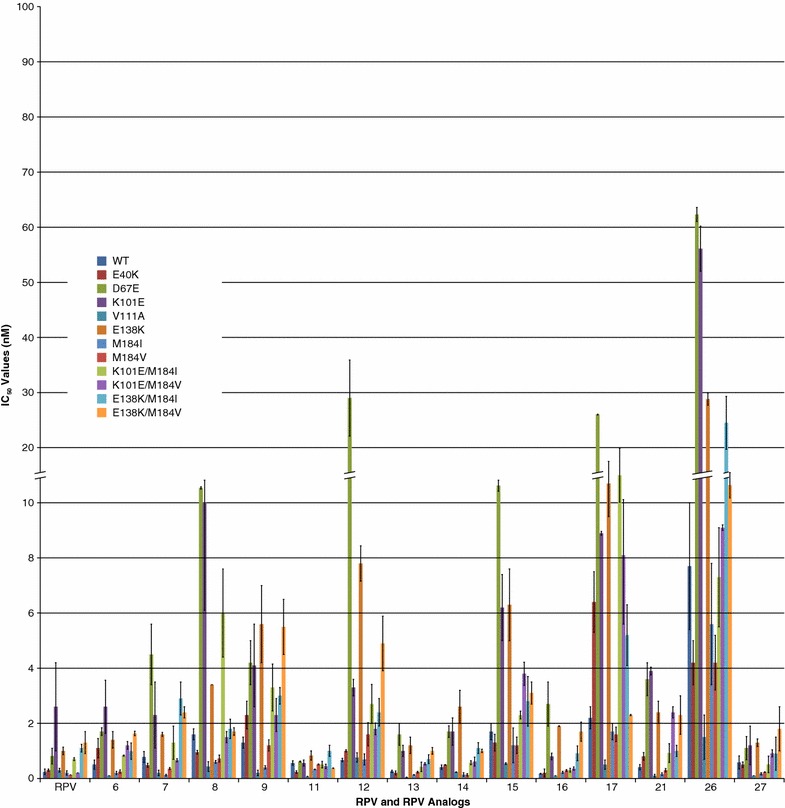
Antiviral activities of **RPV** and **RPV** analogs against mutants selected by **RPV**. The IC_50_ values of **RPV** and the **RPV** analogs were measured against mutants found in **RPV**-treated patients (K101E, E138K, M184I, M184V, K101E/M184I, K101E/M184V, E138K/M184I, and E138K/M184V) or selected by **RPV** in cell culture (E40K, D67E, and V111A) using a single round infection assay. *Error bars* represent the standard deviations of independent experiments, n = 4. The IC_50_ values of the graph are at a maximum value of 100 nM. To better illustrate the lower IC_50_ values, the *Y*-axis is broken from 10 to 20 nM and also shown in the *corresponding bars*

In individuals who fail **RPV**-containing regimens, the E138K mutation is one of the most common NNRTI-resistance mutations. The E138K mutation is frequently associated with M184V or M184I, which are presumably selected by FTC or 3TC, one of which was also present in the regimen [23, 24]. K101E is a resistance mutation that is commonly selected by **RPV** and **ETR**, an NNRTI that is related to **RPV**. In addition, we selected for mutants in cell culture by passaging replication competent HIV-1 in the presence of **11** and obtained viruses that carry additional mutations: E40K, D67E, V111A, E138K, Y181C, and M230I. The selection of viruses carrying mutations at E138K and Y181C was expected because of the contacts made between **11** and the residues of the NNRTI binding pocket; M230I is near the NNRTI binding pocket. Thus, these mutations may interfere with the binding of **11** into the NNRTI binding pocket. The V111A mutation is close in proximity to the triad of aspartic acids (D110, D185, and D186) that comprise the polymerase active site; it is not clear how this mutation could directly affect NNRTI binding. Similarly, the mutations at E40K and D67E are in the fingers subdomain of RT, distant from the NNRTI binding pocket and should not affect NNRTI binding. As discussed earlier, we previously measured the effects of the Y181C and M230L (which should be similar to M230I) mutations on the susceptibility of the vector to these compounds [21]. The RT residues E138 in the RT p51 subunit and K101 of the RT p66 subunit are important because they form a salt bridge at the rim of the NNRTI binding pocket and act as “gate-keepers” that inhibit the entry of NNRTIs into the pocket [25, 26]. The amine linkers of **RPV** make hydrogen bond contacts with the main chain carbonyls of K101 and E138, which help to compensate for the loss of the interaction between the acidic side chains of these two amino acids when **RPV** is bound in the NNRTI binding pocket [27]. To determine if our **RPV** analogs could inhibit the replication of mutants selected in the presence of **RPV**, we tested **RPV** and the **RPV** analogs **6**, **7**, **8**, **9**, **11**, **12**, **13**, **14**, **15**, **16**, **17**, **21**, **26**, and **27** for their ability to inhibit the replication of: E40K, D67E, K101E, V111A, E138K, M184I, M184V, K101E/M184I, K101E/M184V, E138K/M184I, and E138K/M184V (Fig. 4; Additional file 1: Table S3). Although the M184I/V mutations should not directly affect the binding of an NNRTI, some of the viruses selected in patients in trials that included **RPV** and an NRTI had one of these mutations, and we wanted to make sure that the M184I/V mutations did not affect the impact of the K101E or E138K mutations on the susceptibility of the vector to any of the **RPV** analogs. **RPV** and the analogs **6**, **7**, **11**, **13**, **14**, **16**, **21**, and **27** potently inhibited the replication (<2.5 nM) of E40K, D67E, K101E, V111A, E138K, M184I, M184V, K101E/M184I, K101E/M184V, E138K/M184I, and E138K/M184V. The **RPV** analogs **8**, **9**, and **15** were almost as potent (<6 nM) against these mutants, except K101E, which was less susceptible to **8** (10.1 nM ± 4), and D67E, which caused a reduction in susceptibility to both **8** and **15** (12.7 nM ± 0.2 and 13.1 nM ± 1, respectively). **RPV** and the analogs **6**, **13**, **14**, and **27** had comparable antiviral activities against this set of mutants while **11** showed a modestly enhanced susceptibility profile. Analog **17** showed consistently lower potencies against E40K (6.4 nM ± 0.8), D67E (26 nM ± 0.07), K101E (8.9 nM ± 0.07), E138K (13.5 nM ± 4), K101E/M184I (15 nM ± 4.9), and K101E/M184V (8.1 nM ± 2.5). The D67E mutation caused a decrease in susceptibility to the some of the **RPV** analogs **8**, **12**, **15**, and **17**, which is surprising, because, as mentioned earlier, this mutation is not in or near the NNRTI binding pocket. Analog **26** was relatively ineffective against this set of mutants. Overall, it seems that either a pyrimidine or purine is acceptable as a central scaffold as long as amine linkers are present that can interact with K101 of the p66 subunit and E138 of the p51 subunit.

**Fig. 5:**
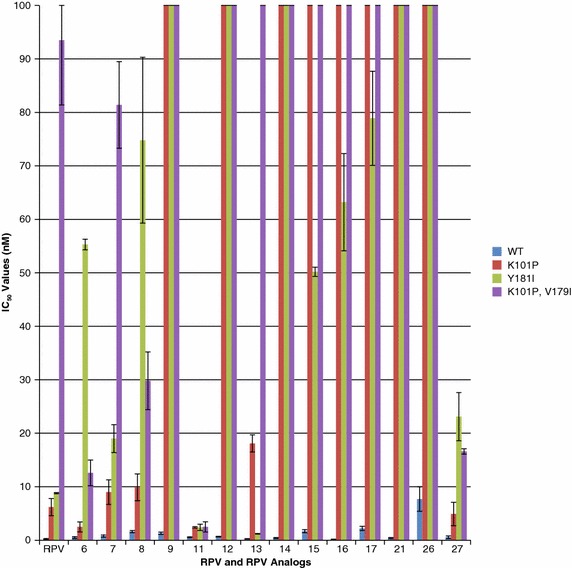
Antiviral activities of **RPV** and the **RPV** analogs against mutants selected by **RPV**. The IC_50_ values for **RPV** and the **RPV** analogs against mutants selected by **RPV** were measured by using a single round infection assay. *Error bars* represent the standard deviations of independent experiments, n = 4. The IC_50_ values of the graph are at a maximum value of 100 nM. The IC_50_ value of several of the **RPV** analogs against the **RPV** resistant mutants were beyond the point of detection in our single round infection assay, >100 nM

In addition to the E138K resistance pathway, there is another **RPV** resistance pathway based on K101P [18, 20]. We tested **RPV** and the **RPV** analogs **6**, **7**, **8**, **9**, **11**, **12**, **13**, **14**, **15**, **16**, **17**, **21**, **26**, and **27** against the mutants K101P, Y181I, and K101P/V179I (Fig. 5; Additional file 1: Table S4). As has already been discussed, we previously tested these compounds against the Y181C mutant [21]. The K101P mutant showed a modest decease in susceptibility to **RPV** (6.2 nM ± 1.6) and the analogs **6** (2.5 nM ± 0.92), **7** (9 nM ± 2.3), **8** (9.9 nM ± 2.5), **11** (2.4 nM ± .14), **13** (18.1 nM ± 1.6), and **27** (4.9 nM ± 2.2) and decreased the susceptibility above the limits of detection in our assay (>100 nM) for the analogs **9**, **12**, **14**, **15**, **16**, **21**, and **26**. The Y181I mutant showed a small reduction in sensitivity to **RPV** (8.8 nM ± 0.12), **11** (2.4 nM ± 0.6), and **13** (1.2 nM ± .06) and showed a more significant loss in susceptibility to **6** (55.3 nM ± 1), **7** (19 nM ± 2.6), **8** (74.8 nM ± 15.5), **15** (50.2 nM ± 0.85), **16** (63.2 nM ± 9.1), **17** (78.9 nM ± 8.8), and **27** (23.1 nM ± 4.5) and a greater loss of susceptibility (>100 nM) for **9**, **12**, **14**, **21**, and **26**. This shows, in terms of susceptibility of the mutants to these compounds, that the impact of the Y181I mutation was considerably greater than the effect of the Y181C mutation [21]. The K101P/Y179I double mutant caused a minor reduction in susceptibility to **6** (12.6 nM ± 2.4), **11** (2.5 nM ± 0.95), and **27** (16.6 nM ± 0.5), a greater decrease in susceptibility to **RPV**, **7**, and **8**, and a large loss of susceptibility (>100 nM) to **9**, **12**, **13**, **14**, **15**, **16**, **17**, **21**, and **26**. As had been true with previous sets of NNRTI-resistant mutants, analog **11** was the most broadly effective in inhibiting the replication of this set of mutants. The K101P/Y179I double mutant was the first mutant we have tested that caused a substantial decrease in susceptibility to **RPV**; this highlights the significance of the fact that this double mutant does not significantly reduce the potency of **11**. The analogs **6** and **27** were also broadly effective against this panel of resistant mutants.

### Modeling the binding of 11 to HIV-1 RT

**Fig. 6:**
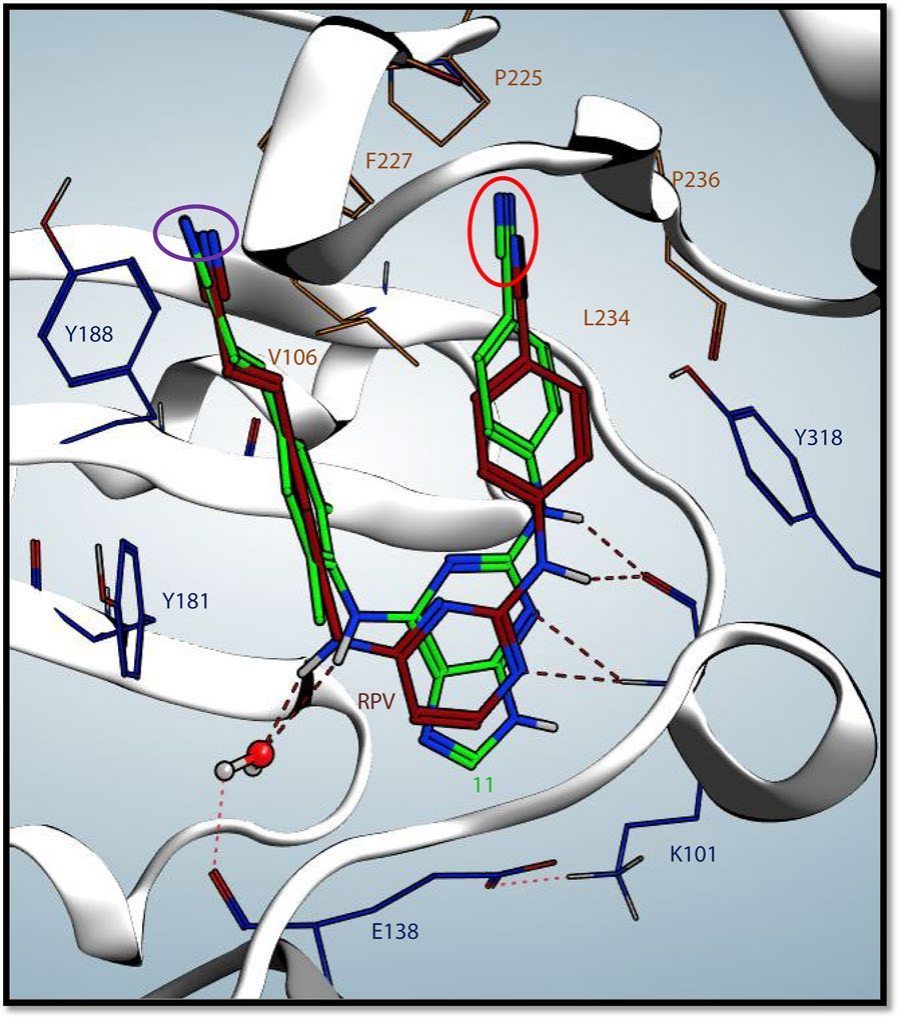
**RPV** and compound **11** in the NNRTI binding pocket. **RPV** (*maroon*) and compound **11** (*green*) are shown bound in the HIV-1 RT NNRTI binding pocket; **11** was docked onto the **RPV** crystal structure. The residues shown in *blue* are important contacts for the binding of **RPV** and denote residues where mutations are selected by the drug. The residues shown in *orange* are important for the interaction of **DOR** with the NNRTI binding pocket; **DOR** selects for mutations at these positions. The *red dashes* represent hydrogen bonds between K101 and **11** and **RPV** and the hydrogen bond network between with water and E138 and **11** and **RPV**. The *purple circle* denotes the difference in the cyanovinyl binding depth between **11** and **RPV**, and the *red circle* shows the difference in the benzonitrile binding depth between **11** and **RPV**

Because of the improved antiviral profile of **11** versus the NNRTI-resistant mutants that we tested, we wanted to understand how it binds to HIV-1 RT, and the contacts it makes in the NNRTI binding pocket (Fig. 6). We docked **11** in the NNRTI binding pocket using the crystal structure of **RPV** bound to HIV-1 RT as the template to model the binding of **11** [28]. The interactions of **11** with the NNRTI binding pocket are similar to the interactions of **RPV** and the pocket, except that the 2,6-purine ring system of **11** resides deeper in the pocket (>1 Å). The benzonitrile linker amine is positioned similarly to the corresponding element of **RPV** and hydrogen bonds with the carbonyl of K101, while the dimethylphenyl linker amine resides in the general vicinity as that of **RPV** (~1 Å away) and most likely interacts with water to form a hydrogen bond network with E138 of the RT p51 subunit. The cyanovinyl that is attached to the dimethylphenyl moiety of **11** is ~0.5 Å deeper in the binding pocket than that of **RPV** (Fig. 6, purple circle), while the benzonitrile moiety of **11** is deeper (~1 Å) in the NNRTI binding pocket compared to **RPV** (Fig. 6, red circle). When **RPV** is modeled into the NNRTI binding pocket of RT with the mutations K101P and Y179I (data not shown), there is steric clash between pyrimidine ring of **RPV** and P101 and I179. It appears that **11** avoids this clash by a slight bend in the central purine ring and by extending the cyanovinyl constituent deeper in the hydrophobic tunnel as well as bending, turning, and extending the benzonitrile moiety towards the back of the NNRTI binding pocket (~2 Å).

**RPV** is the most recent NNRTI to be FDA-approved for the treatment of HIV-1 infection. It was designed and developed to effectively inhibit the replication of a number of common NNRTI-resistant mutants. **RPV** is comprised of three linked rings, and it is smaller than the largest compounds that can bind in the NNRTI binding pocket. As a consequence, it has the ability, when bound to mutant RTs, to undergo conformational changes and shifts in its binding position that allow it to bind tightly to both the WT and mutant forms of RT, altering its conformation to adapt to the changes in the geometry of the binding pocket. However, recent data suggest that **RPV** is susceptible to some NNRTI binding pocket mutations [16–18]. We have made additional **RPV** analogs to determine whether structural changes would allow the new derivatives to be more effective against mutants that reduce the efficacy of **RPV**.

To evaluate the potential of our **RPV** analogs relative to **RPV**, the related drug **ETR**, and **DOR**, we examined antiviral efficacies of the compounds against a broad panel of NNRTI-resistant HIV-1 mutants. Most of the **RPV** analogs exhibited excellent antiviral activities against mutants that were selected by first generation NNRTIs and the new compound **DOR**, which is in late stage clinical trials [19]. In particular, most of the **RPV** analogs displayed strong antiviral activities against mutants that contain E138K. More importantly, a double mutant, K101P/Y179I, which showed a significant drop in susceptibility to **RPV**, remained susceptible to analog **11**, demonstrating that it has an antiviral profile that is broader that any of the NNRTIs we have tested thus far.

Different mutation can arise at position Y181, including Y181I and Y181C. Y181C is susceptible to **RPV** and **RPV** analogs as previously described [21]. However, Y181I caused a substantial decrease in susceptibility to the **RPV** analogs, except for **11** and **13** (2.4 nM ± .6 and 1.2 nM ± .06, respectfully); a minor drop in susceptibility to **RPV** (8.8 nM ± .12) was observed. The isoleucine side chain at position 181 could cause a steric clash with **RPV** and most of the **RPV** analogs that would prevent an interaction with Y183 and the cyanovinyl modification. This interaction is thought to compensate for the disruption of the π-π stacking interaction between the aromatic side chain of Y181 and the phenyl moiety of **RPV** (and most of the **RPV** analogs). The interaction with Y183 can still occur with the Y181C mutant [28].

**11** has a structure that is distinct from the other **RPV** analogs. The central 2,6-purine ring system and the aryl amines of **11** are in a similar conformation to the central pyrimidine ring of the parent compound **RPV**, as opposed to **12**, **13**, **14**, **15**, **16**, **17**, and **21**, which are in a “flipped” conformation. In this arrangement, the cyanovinyl functionality can extend deeper into the hydrophobic tunnel and the benzonitrile moiety can move more towards the back of the NNRTI binding pocket. In addition to the deeper binding of the aryl moieties of **11**, relative to **RPV**, the overall structure and size of **11** could allow it (like **RPV**) to use its torsional flexibility in the NNRTI binding pocket in response to mutations in the NNRTI binding pocket [14, 28].

**Fig. 7:**
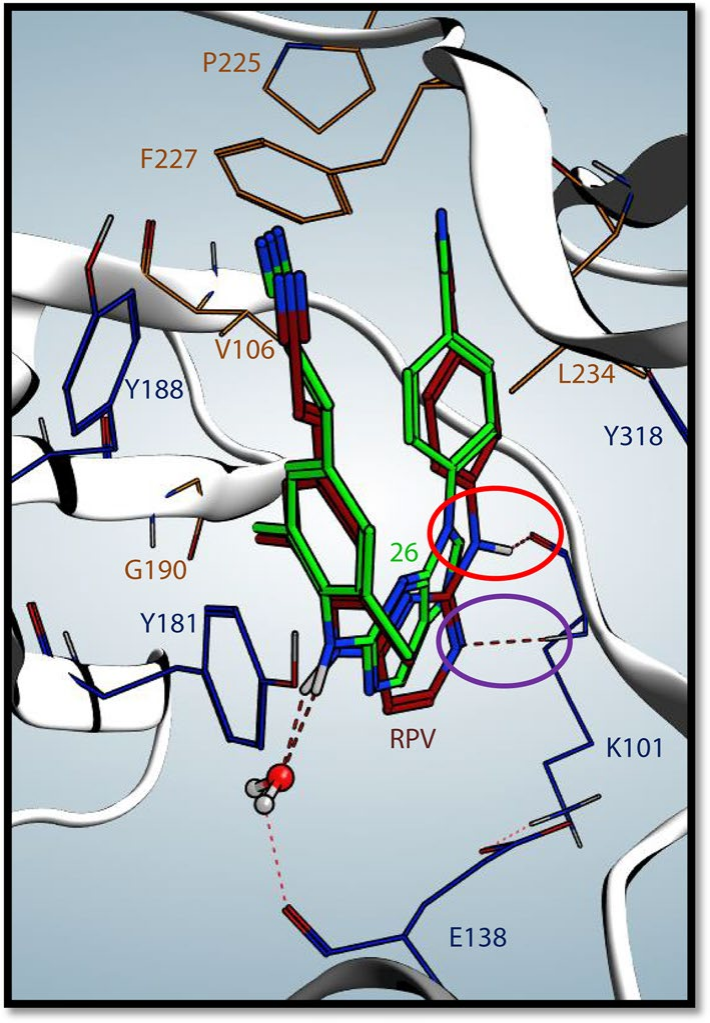
Binding of **RPV** and compound **26** in the NNRTI binding pocket. Using the crystal structure of **RPV** (*maroon*) in the NNRTI binding pocket as a template, **26** (*green*) was docked into the HIV-1 RT NNRTI binding pocket. The residues shown in *blue* are important contacts for the binding of **RPV**; **RPV** selects for mutations at these residues. The residues shown in *orange* are important for the interaction of **DOR** in the NNRTI binding pocket; **DOR** selects for mutations at these positions. The *red circle* shows where amine linker of **RPV** resides, which is a structural feature **26** lacks. Thus **26** fails to interact with main chain carbonyl of K101, suggesting that this interaction, made by **RPV**, is important. The *purple circle* highlights a hydrogen bond interaction between the pyrimidine of **RPV** and the main chain amide of K101, which **26** fails to make, presumably due to its lack of the amine linker. The *red dashes* also depict a hydrogen bond network between water and E138 and **RPV** and **11** of the RT p51 subunit

We also investigated why some of the compounds in the series are much less effective than **RPV** and **11**. Docking **26** into the NNRTI binding pocket (Fig. 7) and comparing its binding to the binding of **RPV**, points to the importance of a hydrogen bond between the amine linker of the **RPV** analogs and the main chain carbonyl of K101 as well as hydrogen bond network between the water molecule, E138 of the RT p51 subunit, and amine linker of **RPV** analogs. **27** is a promising analog that effectively inhibits the double mutant K101P/V179I, which has a reduced susceptibility to **RPV**. It is possible that **27** is effective because of the flexibility of its 2,9-purine ring system and the similarities in the orientation of the central ring to **RPV** and **11**. These two compounds, **11** and **27**, could be used as leads to generate additional modifications that could improve contacts with the NNRTI binding pocket of WT HIV-1 RT and, more importantly, with mutant forms of RT.

## Conclusions

The wide range of NNRTI-resistant mutants that have been identified shows how daunting the challenges are for designing new NNRTIs for the treatment of HIV-1 infection. More and better compounds should be developed to achieve the goal of suppressing the emerging resistant HIV-1 mutants. One possible approach is to identify combinations of two NNRTIs that have non-overlapping resistance profiles and then use them in combination (unpublished observations), rather than trying to prepare a single compound that is effective against the growing array of NNRTI-resistant mutants [29]. Preventing the emergence HIV-1 resistance is a much better and safer treatment strategy than attempting to deal with resistant mutants after they have been selected. However, that strategy does not mean that we should abandon the search for more effective compounds. NNRTIs offer better toxicity profiles than NRTIs and protease inhibitors (PIs) [30, 31]. **11** and **27** represent a promising step in what should be an ongoing process for the development of future NNRTIs. It also appears, based on what is now known, that **DOR**, or perhaps some improved **DOR** derivative, could be useful if it was given in combination with these **RPV** analogs.

## Methods

### Cell-based assays

The human osteosarcoma cell line, HOS, was obtained from Dr. Richard Schwartz (Michigan State University, East Lansing, MI) and grown in Dulbecco’s modified Eagle’s medium (Invitrogen, Carlsbad, CA) supplemented with 5 % (v/v) fetal bovine serum, 5 % newborn calf serum, and penicillin (50 units/ml) plus streptomycin (50 µg/ml; Quality Biological, Gaithersburg, MD). Virion production and single-round infectivity assays were used to determine antiviral activity (IC_50_ values) of the compounds as previously described [32].

### Selection of NNRTI mutations in HIV-1

HuT-CCR5 cells were maintained in RPMI 1640 medium (Life Technologies), 10 % fetal bovine serum (Atlanta Biologicals), 100 U/ml penicillin (Life Technologies), 100 μg/ml streptomycin (Life Technologies), 0.292 mg/ml l-glutamine (Life Technologies), 0.5 μg/ml puromycin (EMD Millipore), and 100 μg/ml G418 (Life Technologies). HuT-CCR5 cells were infected with HIV-1_LAI_ for 2 h at a multiplicity of infection of 0.1–0.01. Cells were cultured in the presence or absence of **RPV**, analog **7**, or analog **11**. Viral RNA from the supernatant was isolated and used as template in RT-PCR assays with primers spanning the RT coding region. PCR product was sequenced to identify any RT mutations.

### Vector constructs

pNLNgoMIVR-ΔEnv.LUC has been described previously [33]. The RT open reading frame was removed from pNLNgoMIVR-ΔEnv.LUC (digestion with SpeI and SalI) and inserted between the SpeI and SalI sites of pBluescript II KS+. Using that construct as the wild-type template, we prepared the following HIV-1 RT mutants using the QuikChange II XL (Stratagene, La Jolla, CA) site-directed mutagenesis protocol kit: L100I, K103N, Y181C, Y188L, H221Y, K103N/Y181C, G190A, G190S, M230L, P236L, L100I/K103N, K103N/P225H, V106A/G190A/F227L, V106A, L234I, V106A/F227L, V106A/L234I, V106A/F227L/L234I, E40K, D67E, K101E, V111A, E138K, M184I, M184V, K101E/M184I, K101E/M184V, E138K/M184I, E138K/M184V, K101P, Y181I, and K101P/V179I. The following sense oligonucleotides were used with matching cognate antisense oligonucleotides (not shown) (Integrated DNA Technologies, Coralville, IA) in the mutagenesis: L100I, 5′-CATCCCGCAGGGATAAAAAAGAAAAAATCA-3′; L100I 2-103, 5′-ATACCACATCCCGCAGGGATTAAAAAGAATAAATCAGTA-3′; K103N, 5′-GCAGGGTTAAAAAAGAATAAATCAGTAACAGTA-3′; Y181C, 5′-CCAGACATAGTTATCTGTCAATACATGGATGAT-3′; Y188L, 5′-TACATGGATGATTTGCTAGTAGGATCTGACTTA-3′; H221Y, 5′-ACACCAGACAAAAAATATCAGAAAGAACCTCCA-3′; G190A, 5′-ATGGATGATTTGTATGTAGCATCTGACTTAGAAATAGGG-3′; G190S, 5′-ATGGATGATTTGTATGTAAGTTCTGACTTAGAAATAGGG-3′; P225H, 5′-AAAAAACATCAGAAAGAACATCCATTCCTTTGGATGGGT-3′; F227, 5′-CATCAGAAAGAACCTCCATTACTTTGGATGGGTTATGAA-3′; M230L, 5′-GAACCTCCATTCCTTTGGCTGGGTTATGAACTCCATCCT-3′; P236L, 5′-ATGGGTTATGAACTGCATCTCGATAAATGGACAGTACAG-3′; V106A, 5′-AAAAAGAAAAAATCAGCAACAGTACTGGATGTG-3′; L234I, 5′-TTCCTTTGGATGGGTTATGAAATCCATCCTGATAAATGGACAGTA-3′; E40K, 5′-GAAATTTGTACAAAAATGGAAAAGGAAGGG-3′; D67E, 5′-GCCATAAAGAAAAAAGAAAGTACTAAATGGAGA-3′; K101E, 5′-CATCCCGCAGGGTTAGAAAAGAAAAAATCAGTAACA-3′; V111A, 5′-GTAACAGTACTGGATGTAGGTGATGCATATTTTTCA-3′; E138K, 5′-CCTAGTATAAACAATAAGACACCAGGGATTAGA-3′; M184I, 5′-GTTATCTATCAATACATAGATGATTTGTATGTA-3′; M184V, 5′-GTTATCTATCAATACGTTGATGATTTGTATGTA-3′; K101P, 5′-CCACATCCCGCAGGGTTACCAAAGAAAAAATCAGTAACA-3′; Y181I, 5′-AATCCAGACATAGTTATCATTCAATACATGGATGATTTG-3′; K101P/V179I, 5′-AAACAAAATCCAGACATAATCATCTATCAATACATGGAT-3′. The double mutants K103N/Y181C, K103N/P225H, and L100I/K103N were made using the previously generated K103N mutant and the appropriate oligonucleotides to add the second mutation, Y181C, P225, and L100I respectively. The double mutants V106A/F227L and V106A/L234I were prepared using the previously generated V106A mutant and the appropriate oligonucleotides to add the second mutation, F227L and L234I, respectively. The double mutants K101E/M184I and K101E/M184V were constructed using the previously generated K101E mutant and the appropriate oligonucleotides to add the second mutation, M184I and M184V, respectively. The double mutants E138K/M184I and E138K/M184V were constructed using the previously generated E138K mutant and the appropriate oligonucleotides for the second mutation, M184I and M184V, respectively. The triple mutant V106A/F227L/L234I was made using the previously generated V106A/F227L double mutant and the appropriate oligonucleotides for the third mutation, L234I. The triple mutant V106A/G190A/F227L was constructed in a series of steps using the previously generated V106A mutant and the appropriate oligonucleotides for the second mutation, G190A, to generate the double mutant V106A/G190A, and then the triple mutant was made using double mutant V106A/G190A and oligonucleotides for the third mutation, F227L. The DNA sequence of each construct was verified by DNA sequence determination. The sequences encoding the mutant RTs were then subcloned into pNLNgoMIVR-ΔEnv.LUC (between the KpnI and SalI sites) to produce the mutant HIV-1 constructs. These DNA sequences were also checked independently by DNA sequence determination.

### Computer modeling

All computer modeling was performed using MOE2014.09 (Chemical Computing Group, Montreal, Quebec, Canada). The previously reported crystal structure of WT HIV-1 RT/**RPV** complex (PDB ID: 2ZD1; [28]) was used in the docking stimulation to model the **RPV** analogs in the NNRTI binding pocket. The docking simulation used a rigid receptor protocol and refinement to predict the pose of the **RPV** analogs in the NNRTI binding pocket. We also used an induced fit docking protocol and refinement that permits the sidechains of the NNRTI binding pocket to move to determine whether this would affect predicted binding of the **RPV** analogs in the NNRTI binding pocket. No new poses were detected.

## Additional file

10.1186/s12977-016-0244-2 The IC_50_ values (nM) of **RPV** and the **RPV** analogs were determined against the NNRTI resistant mutants were measured by using a single round infection assay, n = 4. The concentrations (nM) are measured by reductions in luciferase reporter activity followed by the standard deviations. In parenthesis is the fold-change that describes the difference between the IC_50_ value of the NNRTI resistant mutant relative to WT.
