# Supplementary material for: Rilpivirine analogs potently inhibit drug-resistant HIV-1 mutants
Source: Retrovirology. 2016 Feb 16;13:11. doi: 10.1186/s12977-016-0244-2 (PMC4754833; doi:10.1186/s12977-016-0244-2)
Supplement: Supplementary file 1 — 10.1186/s12977-016-0244-2 The IC50 values (nM) of RPV and the RPV analogs were determined against the NNRTI resistant mutants were measured by using a single round infection assay, n = 4. The concentrations (nM) are measured by reductions in luciferase reporter activity followed by the standard deviations. In parenthesis is the fold-change that describes the difference between the IC50 value of the NNRTI resistant mutant relative to WT. [file 12977_2016_244_MOESM1_ESM.pdf]

Additional file 1: Table S1.

	WT	G190A	G190S	M230L	P236L	L100I/K103N	K103N/P225H	V106A/G190A/F227L
**RPV**	0.24 ± .1	0.4 ± .08 (<2)	0.11 ± .01 (0)	1.7 ± 0 (7)	0.2 ± 0 (0)	2.3 ± .2 (10)	0.6 ± .2 (3)	0.5 ± .02 (2)
**6**	0.5 ± .17	0.2 ± .09 (0)	0.2 ± .09 (0)	3 ± 1.2 (6)	0.23 ± .06 (0)	2.2 ± .6 (4)	0.8 ± .06 (<2)	1.1 ± .2 (2)
**7**	0.78 ± .2	0.3 ± 0 (0)	0.4 ± .07 (0)	4 ± 2 (5)	0.22 ± .06 (0)	1.7 ± .4 (2)	1.1 ± .25 (<2)	1.4 ± .4 (<2)
**8**	1.6 ± .2	0.24 ± .04 (0)	0.17 ± .02 (0)	11.9 ± 1.1 (7)	0.61 ± .07 (0)	5.4 ± .35 (3)	1.2 ± .33 (0)	2.8 ± .42 (<2)
**9**	1.3 ± .2	0.6 ± 0 (0)	0.6 ± .07 (0)	29.6 ± 1.1 (23)	1.7 ± .7 (<2)	44.1 ± 17.6 (34)	4.6 ± .3 (4)	4.6 ± .3 (4)
**11**	0.56 ± .08	0.45 ± .01 (0)	0.2 ± .07 (0)	1.5 ± .5 (3)	0.87 ± .2 (<2)	0.45 ± .01 (0)	0.22 ± .06 (0)	0.98 ± .17 (<2)
**12**	0.67 ± .06	0.9 ± .43 (<2)	0.37 ± .02 (0)	13.8 ± 7.1 (21)	0.8 ± .2 (<2)	4.3 ± 2.6 (6)	3 ± .4 (4)	5.9 ± .14 (9)
**13**	0.26 ± .04	0.3 ± .04 (<2)	0.12 ± 0 (0)	2.2 ± .6 (8)	0.31 ± .02 (<2)	0.58 ± .1 (2)	0.3 ± .09 (<2)	0.5 ± .2 (<2)
**14**	0.41 ± .08	0.13 ± .04 (0)	0.35 ± .05 (0)	2.7 ± .4 (7)	0.15 ± .05 (0)	12.6 ± 1.6 (31)	0.92 ± .14 (2)	0.9 ± .18 (2)
**15**	1.7 ± .3	0.77 ± .19 (0)	0.35 ± .05 (0)	16.8 ± 1.4 (10)	0.9 ± .08 (0)	15.5 ± .6 (9)	1.5 ± .31 (0)	2.6 ± .17 (<2)
**16**	0.17 ± .01	0.37 ± .08 (2)	0.17 ± .05 (0)	2.5 ± 1 (15)	.2 ± 0 (<2)	1.9 ± .7 (11)	2.7 ± .07 (16)	1.7 ± .2 (10)
**17**	2.2 ± .4	1.6 ± .3 (0)	0.74 ± .11 (0)	21.1 ± 8.8 (10)	1.2 ± 0 (0)	63.4 ± 12.6 (29)	3.4 ± .2 (<2)	10.8 ± 2.5 (5)
**21**	0.41 ± .1	0.3 ± .12 (0)	0.2 ± .04 (0)	6.1 ± .14 (15)	0.32 ± .09 (0)	37.6 ± .14 (92)	1.4 ± .07 (3)	3.8 ± 1.6 (9)
**26**	7.7 ± 2.3	2.2 ± .71 (0)	2.1 ± .2 (0)	100	2.91 ± .007 (0)	100	12.9 ± 3.3 (<2)	46.2 ± 11.1 (6)
**27**	0.58 ± .24	0.2 ± .1 (0)	0.1 ± .01 (0)	4.2 ± .5 (7)	0.2 ± .04 (0)	39.8 ± 9.2 (69)	2.1 ± .93 (4)	2.2 ± 1.2 (4)

Antiviral activities (IC_{50} values) of **RPV** and the **RPV** analogs against well-characterized NNRTI resistant mutants were measured by using a single round infection assay, n=4. The concentrations (nM) are measured by reductions in luciferase reporter activity followed by the standard deviations. In parenthesis is the fold-change that describes the difference between the IC_{50} value of the NNRTI resistant mutant relative to WT.

Additional file 1: Table S2.

	WT	V106A	L234I	V106A/F227L	V106A/L234I	V106A/F227L/L234I
**RPV**	0.24 ± .1	0.3 ± .08 (<2)	0.14 ± .06 (0)	0.8 ± .07 (3)	0.1 ± 0 (0)	0.21 ± .01 (0)
**6**	0.5 ± .17	0.31 ± .04 (0)	0.16 ± .08 (0)	2.2 ± .6 (4)	0.24 ± 0 (0)	1.2 ± .14 (2)
**7**	0.78 ± .2	0.4 ± .08 (0)	0.2 ± .1 (0)	1.3 ± .4 (<2)	0.3 ± .1 (0)	0.35 ± .1 (0)
**8**	1.6 ± .2	0.7 ± .13 (0)	0.21 ± .04 (0)	6.3 ± 2 (4)	0.2 ± .1 (0)	2.3 ± .5 (<2)
**9**	1.3 ± .2	2.5 ± 1.2 (<2)	0.4 ± .02 (0)	22.1 ± 2.3 (17)	0.71 ± .09 (0)	6.4 ± 1.8 (5)
**11**	0.56 ± .08	0.22 ± .06 (0)	0.07 ± .01 (0)	0.88 ± .11 (<2)	0.2 ± .08 (0)	3.4 ± 1.1 (6)
**12**	0.67 ± .06	1.1 ± .35 (<2)	0.31 ± .05 (0)	7.6 ± .5 (11)	0.4 ± .03 (0)	1.1 ± .5 (<2)
**13**	0.26 ± .04	0.12 ± .02 (0)	0.2 ± .12 (0)	0.42 ± .03 (<2)	0.1 ± .02 (0)	0.1 ± 0 (0)
**14**	0.41 ± .08	4.3 ± .5 (10)	0.17 ± .04 (0)	4.9 ± 1.3 (12)	1.3 ± .3 (3)	2.7 ± .64 (7)
**15**	1.7 ± .3	2.1 ± .6 (<2)	0.36 ± .08 (0)	1.7 ± .4 (0)	0.19 ± .02 (0)	0.45 ± .06 (0)
**16**	0.17 ± .01	0.53 ± .16 (3)	0.14 ± .04 (0)	0.75 ± .07 (4)	0.1 ± 0 (0)	3 ± .8 (18)
**17**	2.2 ± .4	2.7 ± .64 (2)	0.62 ± .08 (0)	42.8 ± 15.8 (19)	0.71 ± .14 (0)	13.7 ± 6.2 (6)
**21**	0.41 ± .1	1.2 ± .14 (3)	0.4 ± .1 (0)	11.2 ± 3.8 (27)	1 ± .16 (2)	4.3 ± .8 (10)
**26**	7.7 ± 2.3	4.9 ± 1.3 (0)	2.6 ± .71 (0)	65 ± 12.5 (8)	2 ± .4 (5)	35.8 ± 0 (5)
**27**	0.58 ± .24	0.3 ± .08 (0)	0.13 ± .04 (0)	2.9 ± .3 (5)	0.4 ± .2 (0)	0.71 ± .27 (<2)

Antiviral activities (IC_{50} values) of **RPV** and the **RPV** analogs against mutants selected by **DOR** in cell culture were measured by using a single round infection assay, n=4. The concentrations (nM) are measured by reductions in luciferase reporter activity followed by the standard deviations. In parenthesis is the fold-change that describes the difference between the IC_{50} value of the NNRTI resistant mutant relative to WT.

Additional file 1: Table S3.

	WT	E40K	D67E	K101E	V111A	E138K	M184I	M184V	K101E/M184I	K101E/M184V	E138K/M184I	E138K/M184V
**RPV**	0.24 ± .1	0.3 ± .04 (<2)	0.81 ± .28 (3)	2.6 ± 1.6 (11)	0.3 ±.07 (<2)	1 ± .14 (4)	0.2 ± .08 (0)	0.13 ± 0 (0)	0.7 ± .05 (3)	0.2 ± 0 (0)	1.1 ± .14 (5)	1.3 ± .4 (5)
**6**	0.5 ± .17	1.1 ± .35 (2)	1.7 ± .14 (3)	2.6 ± .96 (5)	0.1 ± 0 (0)	1.4 ± .3 (3)	0.2 ± .06 (0)	0.25 ± .06 (0)	0.83 ± .02 (<2)	1.2 ± .14 (2)	0.99 ± .3 (2)	1.63 ± .08 (3)
**7**	0.78 ± .2	0.48 ± .07 (0)	4.5 ± 1.1 (6)	2.3 ± 1.2 (3)	0.2 ± .1 (0)	1.6 ± .07 (2)	0.12 ± .03 (0)	0.36 ± .04 (0)	1.3 ± .6 (<2)	0.66 ± .06 (0)	2.9 ± .6 (4)	2.4 ± .2 (3)
**8**	1.6 ± .2	0.95 ± .07 (0)	12.7 ± .2 (8)	10.1 ± 4 (6)	0.43 ± .18 (0)	3.4 ± 0 (2)	0.6 ± .05 (0)	0.72 ± .13 (0)	6 ± 1.6 (4)	1.5 ± .2 (0)	1.8 ± .35 (<2)	1.7 ± .14 (<2)
**9**	1.3 ± .2	2.3 ± .5 (2)	4.2 ± .8 (3)	4.1 ± 1.5 (3)	0.2 ± .1 (0)	5.6 ± 1.4 (4)	0.4 ± .06 (0)	1.2 ± .2 (0)	3.3 ± .85 (3)	2.3 ± .6 (<2)	3 ± .3 (2)	5.5 ± 1 (4)
**11**	0.56 ± .08	0.23 ± .05 (0)	0.61 ± .02 (<2)	0.56 ± .11 (0)	0.07 ± .007 (0)	0.83 ± .17 (<2)	0.33 ± .007 (0)	0.51 ± .02 (0)	0.5 ± .12 (0)	0.44 ± .08 (0)	1 ± .2 (<2)	0.37 ± .007 (0)
**12**	0.67 ± .06	1 ± .04 (<2)	29 ± 6.9 (43)	3.3 ± .3 (5)	0.76 ± .17 (<2)	7.8 ± .64 (12)	0.69 ± .2 (0)	1.6 ± .42 (2)	2.7 ± .71 (4)	1.8 ± .21 (3)	2.4 ± .5 (4)	4.9 ± .99 (7)
**13**	0.26 ± .04	0.2 ± .07 (0)	1.6 ± .4 (6)	1 ± .2 (4)	0.04 ± .01 (0)	1.2 ± .3 (5)	0.13 ± .01 (0)	0.23 ± .03 (0)	0.42 ± .17 (<2)	0.53 ± .04 (2)	0.7 ± .16 (3)	1 ± .12 (4)
**14**	0.41 ± .08	0.49 ± .01 (<2)	1.7 ± .21 (4)	1.7 ± .5 (4)	0.23 ± .01 (0)	2.6 ± .6 (6)	0.14 ± .07 (0)	0.13 ± .04 (0)	0.57 ± .07 (<2)	0.61 ± .16 (<2)	1.1 ± .2 (3)	1 ± .06 (2)
**15**	1.7 ± .3	1.3 ± .3 (0)	13.1 ± 1 (8)	6.2 ± 1.2 (4)	0.54 ± .03 (0)	6.3 ± 1.3 (4)	1.2 ± .63 (0)	1.2 ± .3 (0)	2.3 ± .14 (<2)	3.8 ± .42 (2)	2.8 ± .9 (<2)	3.1 ± .4 (<2)
**16**	0.17 ± .01	0.2 ± .14 (<2)	2.7 ± .8 (16)	0.8 ± .12 (5)	0.1 ± 0 (0)	1.9 ± .01 (11)	0.22 ± .04 (<2)	0.29 ± .03 (<2)	0.3 ± .07 (<2)	0.36 ± .06 (2)	0.91 ± .27 (5)	1.7 ± .35 (10)
**17**	2.2 ± .4	6.4 ± .8 (3)	26 ± .07 (12)	8.9 ± .07 (4)	0.5 ± .17 (0)	13.5 ± 4 (6)	1.7 ± .3 (0)	1.6 ± .26 (0)	15 ± 4.9 (7)	8.1 ± 2.5 (4)	5.2 ± 1.1 (2)	2.3 ± .02 (<2)
**21**	0.41 ± .1	0.8 ± .1 (2)	3.6 ± .6 (9)	3.9 ± .14 (10)	0.1 ± .05 (0)	2.4 ± .4 (6)	0.16 ± .05 (0)	0.3 ± .06 (0)	0.92 ± .34 (2)	2.4 ± .2 (6)	1 ± .2 (2)	2.3 ± .7 (6)
**26**	7.7 ± 2.3	4.2 ± .8 (0)	62.3 ± 1.3 (8)	56.1 ± 4.1 (7)	1.5 ± .8 (0)	28.8 ± 1.1 (4)	5.6 ± 2.2 (0)	4.2 ± .99 (0)	7.3 ± 1.8 (0)	9.1 ± .1 (<2)	24.5 ± 4.8 (3)	13.2 ± 2.3 (<2)
**27**	0.58 ± .24	0.5 ± .1 (0)	1.1 ± .42 (2)	1.2 ± .7 (2)	0.1 ± 0 (0)	1.3 ± .14 (2)	0.17 ± .04 (0)	0.23 ± .007 (0)	0.51 ± .3 (0)	0.91 ± .13 (<2)	0.9 ± .6 (<2)	1.8 ± .8 (3)

Antiviral activities (IC_{50} values) of **RPV** and the **RPV** analogs against mutants selected by **RPV** were measured by using a single round infection assay, n=4. The concentrations (nM) are measured by reductions in luciferase reporter activity followed by the standard deviations. In parenthesis is the fold-change that describes the difference between the IC_{50} value of the NNRTI resistant mutant relative to WT.

Additional file 1: Table S4.

	WT	K101P	Y181I	K101P, V179I
**RPV**	0.24 ± .1	6.2 ± 1.6 (26)	8.8 ± .12 (37)	93.5 ± 12.1 (390)
**6**	0.5 ± .17	2.5 ± .92 (5)	55.3 ± 1 (110)	12.6 ± 2.4 (25)
**7**	0.78 ± .2	9 ± 2.3 (12)	19 ± 2.6 (24)	81.4 ± 8.1 (104)
**8**	1.6 ± .2	9.9 ± 2.5 (6)	74.8 ± 15.5 (47)	29.8 ± 5.4 (19)
**9**	1.3 ± .2	100	100	100
**11**	0.56 ± .08	2.4 ± .14 (4)	2.4 ± .6 (4)	2.5 ± .95 (4)
**12**	0.67 ± .06	100	100	100
**13**	0.26 ± .04	18.1 ± 1.6 (70)	1.2 ± .06 (5)	100
**14**	0.41 ± .08	100	100	100
**15**	1.7 ± .3	100	50.2 ± .85 (30)	100
**16**	0.17 ± .01	100	63.2 ± 9.1 (372)	100
**17**	2.2 ± .4	100	78.9 ± 8.8 (36)	100
**21**	0.41 ± .1	100	100	100
**26**	7.7 ± 2.3	100	100	100
**27**	0.58 ± .24	4.9 ± 2.2 (8)	23.1 ± 4.5 (40)	16.6 ± .5 (29)

Antiviral activities (IC_{50} values) of **RPV** and the **RPV** analogs against mutants selected by **RPV** were measured by using a single round infection assay, n=4. The concentrations (nM) are measured by reductions in luciferase reporter activity followed by the standard deviations. In parenthesis is the fold-change that describes the difference between the IC_{50} value of the NNRTI resistant mutant relative to WT.
